# Test-retest reliability of template-defined vs. manual tracing processing streams in neuromelanin-sensitive magnetic resonance imaging analysis

**DOI:** 10.1016/j.ynirp.2025.100290

**Published:** 2025-09-26

**Authors:** Tyler A. Lesh, Jason Smucny, Joshua P. Rhilinger, Sarvenaz Pakzad, Guillermo Horga, Cameron S. Carter

**Affiliations:** a Department of Psychiatry and Behavioral Sciences, University of California, Davis, 1 Shields Ave, Davis, CA, 95616, USA; b Department of Psychiatry, Columbia University, 116th and Broadway, New York, NY, 10027, USA; c Department of Psychiatry and Human Behavior, University of California, Irvine, 401 E. Peltason Drive, Irvine, CA, 92617, USA

**Keywords:** Dopamine, MRI, Neuromelanin, Psychosis, Schizophrenia, Substantia nigra

## Abstract

Neuromelanin (NM) magnetic resonance imaging (MRI) is a relatively new, noninvasive method used as a proxy measure of midbrain dopamine function. Previous studies in schizophrenia have found evidence of enhanced signal in patients that are associated with positive psychotic symptoms. However, there are limited data available comparing methods of computing signal in substantia nigra (SN). We sought to examine the reliability and validity of manual tracing vs template-defined methods to help guide the field in identifying optimal approaches in NM imaging. NM-MRI was performed on 22 participants (18 with early psychosis (EP) and 4 healthy controls (HCs)) scanned twice over a 1–14 week period. Mean SN NM signal was calculated using template-defined and manual tracing methods to define SN ROIs. Intraclass correlation coefficients (ICCs) based on absolute agreement were calculated between test and retest. Correlations between NM signal for each method and symptoms in EP were also examined. ICCs for the template-defined were in the excellent range (.81–.85) and manual tracing methods were in the poor to fair range (−.14 to .56). The template-defined method showed a trend positive relationship with reality distortion symptoms (r = .45, *p* = .06) and the manual tracing method showed a significant positive relationship (r = .47, *p* = .05). Supplemental analyses highlighted the importance of thresholding and using the mode to compute CNR for identifying these relationships to symptomatology. While both methods showed clinical validity, the excellent reliability of the template-defined method suggests this technique is the preferred strategy for NM-MRI analysis.

## 1 Introduction

The ability to measure dopamine in the living human brain has long held the interest of researchers and clinicians alike. For decades, this was only possible through positron emission tomography (PET), which requires the use of expensive, invasive, radioactive tracers. The advent of neuromelanin (NM)-based magnetic resonance imaging (MRI), however, has opened a new window of opportunity as it enables measurement of a dopamine proxy without needing a PET scanner or any radioactive injections (Horga et al., 2021).

NM is a dark pigment that accumulates in the substantia nigra (SN) and locus coeruleus as a product of iron-dependent dopamine oxidation (Zecca et al., 2008). NM accumulates over the lifespan, and due to its paramagnetic properties NM-pigmented neuronal bodies can be measured using NM-MRI sequences sensitive to these properties. Validating its use as a dopaminergic marker, previous work found a significant correlation between striatal presynaptic dopamine release capacity (as measured by PET) and NM signal in the SN (Cassidy et al., 2019; Vano et al., 2024). Cassidy and colleagues (Cassidy et al., 2019) also found that NM-MRI signal correlated with NM concentration and SN activity in postmortem tissue, further validating the measure. Providing additional clinical validation for the measure, studies suggest NM-MRI signal is decreased in Parkinson's disease (a disorder caused by degeneration of dopaminergic neurons) (Yan et al., 2024) and increased in psychosis (a condition associated with hyperdopaminergia) (Horga et al., 2021). Related to this point, even though hyperdopaminergia is associated with psychosis, it might not occur in all individuals that present with psychotic symptoms. Indeed, it is hypothesized that people with "treatment-resistant” schizophrenia (in which first line antipsychotic treatments are ineffective) may have a non-dopaminergic basis (Potkin et al., 2020).

To measure NM-MRI intensity at a region-of-interest (ROI) level, there are multiple viable strategies. Early techniques (Sasaki et al., 2006) focused on placing circular cursors in the SN and adjacent reference regions to compute a contrast to noise ratio. Other, more recent techniques ((e.g., Chen et al., 2014; Schwarz et al., 2011)) involve manually tracing the rough outline of the SN and then using signal intensity differences between the SN and reference regions to further threshold and refine the ROI. One potential drawback of these techniques is that even with adequate training, there may be inter-rater biases introduced with different subjective thresholds as to what may be considered sufficient signal intensity to outline the region. Additionally, due to the relatively low spatial resolution in the slice dimension, there can be variability in how the most ventral portion of the SN is captured and where a rater may decide to begin manually tracing. While some other strategies involve using semi-automated algorithms to expand the region of interest from a starting point (e.g., Ogisu et al., 2013; Reimao et al., 2015) these often still rely upon manual intervention to select the reference and/or seed regions. One alternative to these techniques is to use a template-defined ROI approach that relies upon accurate normalization of the pre-defined MNI-space template to each individual subject. Prior work suggests this method has good to excellent test-retest reliability (Cassidy et al., 2019; Langley et al., 2017; van der Pluijm et al., 2021; Wengler et al., 2020) although to our knowledge no study has yet evaluated the reliability of both the manually-defined and template-defined testing methods in the same sample. To that end, we examined intraclass correlation coefficients (ICCs) for each method in a sample of healthy control (HC) individuals and people with early psychosis (EP).

## 2 Methods

### 2.1 Sample

NM imaging test-retest data were available for 18 participants with EP (“EPs”; six with a schizophrenia-spectrum diagnosis (schizophrenia, schizoaffective, and schizophreniform disorder), six with other specified psychotic disorder, four with type I bipolar disorder (BD) with psychotic features, two with major depressive disorder with psychotic features) and four healthy controls (HCs). EPs were recruited as outpatients from the University of California, Davis (UCD) Early Diagnosis and Preventive Treatment (of Psychosis) (EDAPT) research clinic (http://earlypsychosis.ucdavis.edu). Treatment in the clinic follows a coordinated specialty care (CSC) for early psychosis model delivered by an interdisciplinary treatment team. Treatment includes detailed clinical assessments using gold-standard structured clinical interviews and medical evaluations, targeted pharmacological treatments including low dose atypical antipsychotic treatment, individual and family-based psychosocial education and support, cognitive behavioral therapy for psychosis, and support for education and employment. The Structured Clinical Interview for DSM-5 (SCID) (First et al., 2016) was used for diagnosis of psychopathology. Diagnoses were confirmed by a group of trained clinicians during case-conferences. All EP participants reported psychosis onset within two years of the date of informed consent. Individuals were excluded for a diagnosis of major medical or neurological illness, head trauma, Weschler Abbreviated Scale of Intelligence-2 score (WASI-2) (Weschler, 1999) score <70, and magnetic resonance imaging (MRI) exclusion criteria (e.g. claustrophobia, metal in the body). Individuals were also excluded for substance abuse within the previous three months (including positive urinalysis on the day of scanning) although recreational cannabis use was allowed. Recreational cannabis users were not permitted to use cannabis on the day of scanning. HC were excluded based on the presence of current or past DSM-5 diagnosis as well as presence of first-degree relatives with a psychotic disorder. All participants provided written informed consent and were compensated for participation. The UCD Institutional Review Board approved the study. Medication regimen (type and chlorpromazine (CPZ)-equivalent dose) was assessed by clinical records at baseline and follow-up.

Symptoms in EPs were assessed using the Brief Psychiatric Rating Scale (BPRS) (Ventura et al., 1993), Scale for the Assessment of Negative Symptoms (SANS) (Andreasen, 1984a) and Scale for the Assessment of Positive Symptoms (SAPS) (Andreasen, 1984b). Consistent with prior work (Barch et al., 2003), three core symptom dimensions were computed: Poverty, Disorganization, and Reality Distortion. “Poverty” was calculated as the sum of emotional withdrawal, motor retardation, and blunted affect from the BPRS with anhedonia/asociality, avolition/apathy, alogia, and affective flattening from the SANS. “Disorganization” was calculated as the sum of conceptual disorganization, mannerisms and posturing, and disorientation scores from the BPRS, the attention score from the SANS, as well as positive formal thought disorder and bizarre behavior scores from the SAPS. “Reality distortion” was calculated as the sum of grandiosity, suspiciousness, hallucinations, and unusual thought content from the BPRS with hallucinations and delusions from the SAPS.

### 2.2 MRI acquisition

Participants were scanned twice with an inter-scan interval ranging from 1 week to 14 weeks. T1 weighted MPRAGE structural images were acquired for realignment and normalization during preprocessing. Structural imaging parameters were 2530 ms TR, 3.5 ms TE, flip-angle 7°, 256 mm^2^ FOV, 1 mm isotropic voxels.

Individual NM images were acquired on one of three 3 T MR scanners (Siemens Tim Trio (n = 7), Skyra (n = 3), and Prisma (n = 12)) using a 32-channel head coil. Test and retest images were always acquired on the same scanner. The first participants of the study were scanned on the Tim Trio. While the Trio was undergoing a planned upgrade to the Prisma Fit, three participants were scanned on the Skyra. All remaining participants were scanned on the upgraded Prisma scanner. The base neuromelanin-sensitive pulse sequence was acquired from Dr. Xiaoping Hu (Chen et al., 2014) and slightly modified to collect thinner slices in the z-axis per recommendations by Wengler et al. (2020). The number of sequential measurements was also increased to further improve SNR. Sequence parameters were as follows: 18.5 min 2D GRE with MTC pulse at 300° flip angle, 13 sequential measurements, 387 ms TR, 4.03 ms TE, 25° flip angle, 200 mm FoV, 0.4 mm × 0.4 mm in-plane resolution. Fourteen slices were acquired, and the slice thickness was set to 2 mm for 18 participants and 2.5 mm for four participants. Adjustment of the slice thickness for these four individuals was necessary to keep SAR values within range.

### 2.3 NM MRI preprocessing

NM images were preprocessed using two distinct pipelines: one with a manually traced mask definition and one with template-defined mask alignment. All volumes collected were visually inspected for the presence of motion and other artifacts before beginning either pipeline. Volumes with poor image quality were excluded from analysis in both pipelines.

The manual mask tracing method closely approximated the procedures of Chen et al. (2014) and also shares some features developed by Schwarz et al. (2011). Briefly, good quality volumes were aligned to the mean volume via rigid body registration (FSL's mcflirt), and an average image was generated. One of two raters (JPR = 15 participants and SP = 7 participants) were assigned to process both the test and retest scans of a given participant. Raters were trained to identify the substantia nigra (SN) and draw a mask of the SN within two to three slices in the participant's native space. Fslview was used to visualize and draw masks and image intensity thresholds were adjusted to maximize the contrast of the SN compared to surrounding tissue. Raters started by identifying the most ventral slice in which the SN could be visualized and then attempted to trace up to three total slices. Circular ROIs (diameter = 3.2) were placed in the crus cerebri (CC) of each selected slice. The mean voxel intensity and standard deviation were calculated for all CC ROIs combined. The SN mask for was then thresholded such that mask voxels were removed if the voxel values were lower in intensity than the average CC value plus two standard deviations. SN contrast to noise ratio (CNR) images were then calculated by subtracting the mean value of the CC from voxels in the refined (thresholded) SN mask and dividing by the standard deviation of the CC. The mean CNR value was computed across all voxels in the SN that were greater than zero.

The template-defined mask alignment pipeline was programmed in MATLAB and followed procedures reported by Cassidy et al. (2019). Briefly, rigid body transformation was used to realign included NM volumes to the first image and generate an average image. Each participant's average NM image was then aligned to their individual T1-weighted structural image via rigid body registration. An Advanced Normalization Tools (ANTS) (Tustison et al., 2014) deformable registration was used to align the T1 image to a template image in MNI space, and the same diffeomorphic transformation was applied to the average NM image to carry it into MNI space. Average NM images were resliced to 1 mm isotropic voxels and AFNI was then used to smooth the NM image with a 1 mm Gaussian kernel. A CNR image was generated by subtracting the mode of the distribution of voxels within a pre-existing reference region mask (CC) from the average image, dividing by the mode of the CC, and multiplying by 100. The mode was defined by a kernel-smoothing function fit to the histogram of all voxels within the CC mask for each participant. A pre-existing SN mask was then applied to the CNR image, and the mean CNR value within the mask was calculated.

In order to better compare these two methodologies, two supplemental analyses were performed. First, the manual mask tracing pipeline was modified to compute voxelwise CNR using calculations consistent with that performed in the template-defined pipeline (i.e., subtracting the mode of the CC from voxels within the SN mask and dividing by the mode of the CC). Second, the template-defined pipeline was modified to include an additional “mask refinement” step consistent with that used in the manual mask pipeline (i.e., removing SN voxels that were below the mean plus two standard deviations of the CC mask). By considering these additional analyses, the mask refinement and mathematical operations are largely matched between the two pipelines, with the main difference largely isolated to the manner in which the masks are created and aligned to the data.

**Fig. 1:**
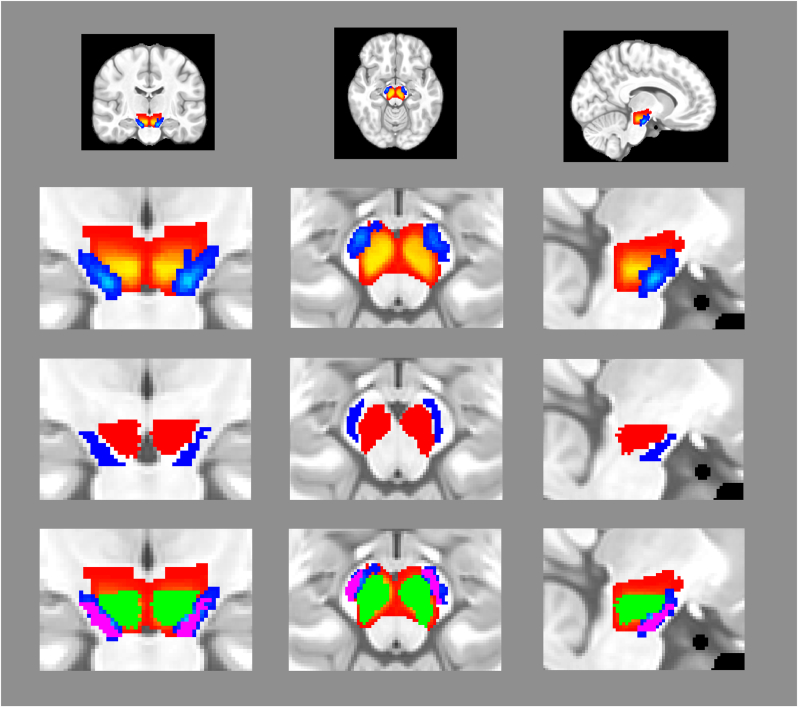
**Top Row:** Coronal, axial, and sagittal view of manually-traced substantia nigra (red to yellow spectrum) and crux cerebri (blue to light blue spectrum) masks displayed on whole-brain MNI template. **Second Row:** Zoomed-in view of manually-traced masks from top row. The brighter the color spectrum (i.e. more yellow or light blue) of a given voxel indicates a greater number participants who contain that voxel in their mask. **Third Row:** Zoomed-in view of template-defined substantia nigra (red) and CC (blue) masks. **Bottom Row:** Zoomed-in view of manually-defined and template-defined masks. Voxels that are overlapping between the two processing streams are presented in green (substantia nigra) and violet (crux cerebri).

To visually compare the anatomical placement and overlap of the manually-defined and template ROIs, the SN and CC masks for each session of each participant were brought into the MNI template space by using the same diffeomorphic transformation computed by the template-defined pipeline for that session and participant. These masks were resampled to 1 mm isotropic and were slightly eroded to mitigate the effects of interpolation due to resampling. Resampled masks were then binarized and summed across sessions to demonstrate the number of sessions in which each voxel was included in the manually-defined SN and CC masks (Fig. 1).

### 2.4 Data harmonization

Because three different scanners were used, scanner effects were regressed out using the ComBat toolbox (Fortin et al., 2018) using parametric settings.

### 2.5 Test-retest reliability

Intraclass Correlation Coefficients (ICCs) and their 95 % confidence intervals were calculated using the SPSS statistical package version 29 (SPSS Inc, Chicago, IL) based on a mean-scan (k = 2), absolute-agreement, two-way mixed-effects model. This model was selected based on work summarized by Koo and Li (2016) which emphasizes the mixed-model suitability due to the fact that the repeated scans cannot be considered random samples. ICCs greater than .75 were considered "excellent”, .60 to .75 ″good”, .40 to .60 ″fair,” and less than .40 ″poor” (Cicchetti, 1994).

## 3 Results

### 3.1 Participants

Demographic and clinical data for participants are presented in Table 1. Data from 22 individuals were available for test-retest analysis (4 HCs and 18 EPs). An average of 25 days elapsed between test and retest NM-MRI scans (min = 5 days, max = 104 days).

**Table 1:** Demographic information for participants included in primary analyses. Numbers in parentheses represent the standard deviation. Abbreviations: BD = Bipolar Disorder, CPZ = Chlorpromazine, EP = Early Psychosis, HC = Healthy Controls, WASI-2 = Weschler Abbreviated Scale of Intelligence, 2nd Edition. Parental education data were unavailable for 4 individuals with EP. WASI-2 score was unavailable for 1 HC. Medication data were unavailable for 2 individuals with EP.

	HC (*n* = 4)	EP (*n* = 18)	All (*n* = 22)
**Age**	23.08 (1.40)	20.25 (3.11)	20.76 (3.06)
**Sex (M/F)**	0/4	7/11	7/15
**Handedness (L/R/Both)**	0/4	0/17/1	0/21/1
**Education Level (Years)**	15.50 (1.29)	12.28 (1.99)	12.86 (2.25)
**Parental Education Level (Years)**	13.50 (2.80)	15.00 (2.94)	14.67 (2.90)
**IQ (WASI-2)**	115.00 (10.58)	106.33 (14.06)	107.57 (13.74)
**Days between Test and Retest**	7.00 (1.63)	28.83 (25.29)	24.86 (24.34)
***N* Medicated/Unmedicated**	–	9/7	9/11
**Mean Antipsychotic Dose in CPZ Equivalents**	–	146.24 (239.24)	146.24 (239.24)
**Reality Distortion Symptoms Score**	–	12.28 (7.73)	12.28 (7.73)
**Poverty Symptoms Score**	–	13.50 (9.79)	13.50 (9.79)
**Disorganization Symptoms Score**	–	8.61 (4.47)	8.61 (4.47)

### 3.2 Neuromelanin test-retest analysis

**Fig. 2:**
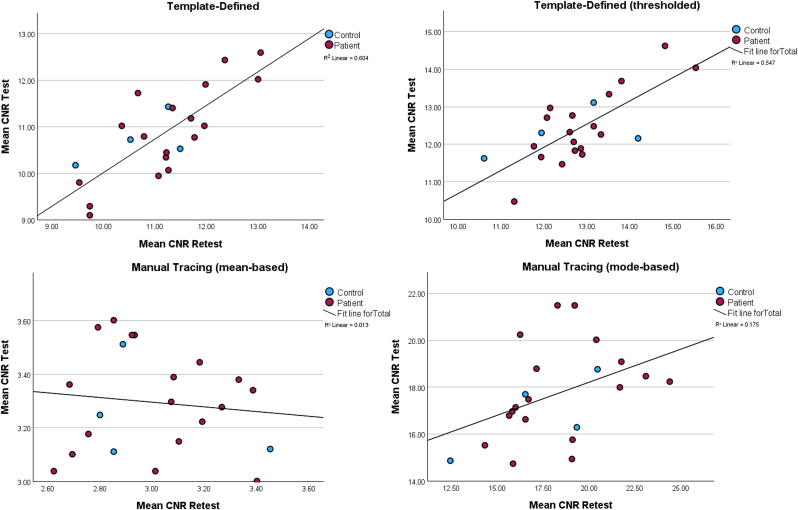
**Upper Left:** Scatterplot showing the relationship between test and retest neuromelanin (NM) contrast to noise ratio (CNR) for the template-defined method. **Upper Right:** Scatterplot showing the relationship between test and retest NM CNR for the template-defined method using additional thresholding. **Lower Left:** Scatterplot showing the relationship between test and retest NM CNR for the manual tracing method using the mean. **Lower Right:** Scatterplot showing the relationship between test and retest NM CNR for the manual tracing method using the mode. Early psychosis (EP) individuals are represented as red circles and healthy control (HC) individuals as blue circles. Linear regression lines are plotted across each sample.

Results of test-retest analyses for NM segregated by analysis pipeline are presented in Table 2, with scatterplots showing the relationship between test and retest NM signals for each method shown in Fig. 2 (coded by diagnosis) and Supplementary Fig. 1 (coded by scanner used). Briefly, across all participants the template-defined tracing method showed excellent NM CNR test-retest reliability (ICC = .85, 95 % CI = .63–.94, Fig. 2 upper left). Reliability was similarly high when adding an additional processing step to threshold the SN mask based upon the mean and standard deviation of the CC (ICC = .81, 95 % CI = .52 - .93, Fig. 2 upper right). In contrast, the manual tracing method showed poor test-retest reliability (ICC = −.14, 95 % CI = −.75 – .38, Fig. 2 lower left) when computing CNR based upon the mean and standard deviation of the CC. Computing CNR based upon the mode of the reference region dramatically improved the reliability of the manual tracing method (ICC = .56, 95 % CI = −.05–.82, Fig. 2 lower right). Fig. 1 illustrates the anatomical coverage and comparability between the manually-defined and template-defined ROIs. Although a minority of participants showed voxels outside of the template region (dark red voxels), a high degree of overlap was observed between both ROIs for most participants (green and violet regions in bottom row).

**Table 2:** Harmonized neuromelanin (NM) data. Numbers in parentheses represent the standard deviation unless otherwise specified. Abbreviations: CI = Confidence Interval, ICC = Intraclass Correlation Coefficient. ICC values were calculated based on a mean-scan (k = 2), absolute-agreement, two-way mixed-effects model. ∗*p* < .05.

Method	Test	Retest	ICC (95 % CI)
**Template-Defined**	10.85 (.94)	11.16 (1.01)	.85 (.63–.94)∗
**Template-Defined (thresholded)**	12.43 (.94)	12.83 (1.12)	.81 (.52–.93)∗
**Manual Tracing**	3.30 (.19)	3.01 (.25)	−.14 (−.75–.38)
**Manual Tracing (mode)**	17.70 (2.0)	18.19 (2.9)	.56 (−.05 – .82)∗

### 3.3 Relationships to symptoms

**Fig. 3:**
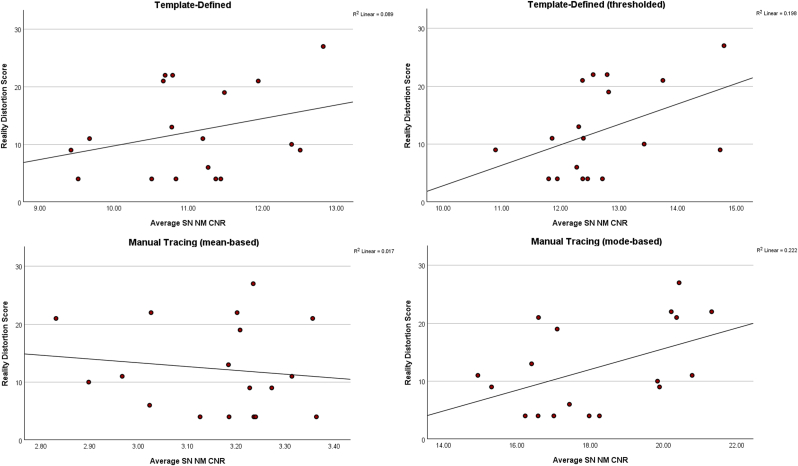
**Left:** Scatterplot showing the relationship between the average test/retest neuromelanin (NM) contrast to noise ratio (CNR) and reality distortion symptoms for the template-defined (**Upper Left**), template-defined with additional thresholding (**Upper Right**), manual tracing based on the mean (**Lower Left**), and manual tracing based on the mode (**Lower Right**).

Scatterplots showing relationships between mean NM CNR across test and retest and reality distortion symptoms are shown in Fig. 3. Briefly, reality distortion symptoms were positively related to the average NM CNR when NM levels were calculated using the template-defined method (albeit non-significantly) (*r* = .30, *p* = .24). Notably, when thresholding the template-defined mask, the resulting data showed a strong and trend-level relationship with reality distortion symptoms (r = .45, p = .06). The relationship between reality distortion symptoms and the NM CNR calculated using the manual tracing method, however, was in the opposite direction (*r* = −.13, *p* = .61). Use of the mode in calculating CNR for the manual tracing method revealed a significant positive relationship with reality distortion (r = .47, p = .05). To determine whether NM correlations were specific to symptoms associated with hyperdopaminergia, we also computed correlations with disorganization and poverty symptoms (see Supplementary Table 1). Poverty and disorganization symptoms were not significantly associated with the template-defined method (all p > .3), although disorganized symptoms were significantly positively associated with NM CNR based upon the manual tracing method using the mode (r = .55, p = .02).

## 4 Discussion

Test-retest reliability values were excellent for the template-defined method but were in the poor to fair range for the manual tracing method. Notably, when utilizing similar mask refinement and CNR calculations based upon the mode of reference regions, both methodologies revealed associations with reality distortion symptoms, such as hallucinations and delusions. While both techniques showed evidence of clinical validity, the additional identified relationships with disorganized symptoms (e.g., thought disorder and attention) in the mode-based manual tracing method show less specificity for symptoms thought to be associated with hyperdopaminergia. Overall, these results revealed that NM processing procedures are critically important for obtaining reliable, accurate, and clinically meaningful output.

The excellent reliability values observed here are consistent with previous NM-MRI studies using template-defined methods to define ROIs (Cassidy et al., 2019; Langley et al., 2017; van der Pluijm et al., 2021; Wengler et al., 2020). Taken together with our finding of poor to fair reliability using the manual tracing method, these results suggest that the template-defined pipeline should be prioritized for NM-MRI clinical and research applications. The template-defined method also circumvents the inherent "circularity” endemic to the manual tracing method, in that the latter method uses NM signal to define ROI masks (Wengler et al., 2024b). Remarkably, the observed reliability for the template-defined method was quite robust considering that three different scanners were used over the course of the study (although the same scanner was used for test and retest scans for each individual), illustrating the power of the template-defined pipeline.

Notably, the reliability of the manual tracing method was much lower than initially hypothesized. In fact, when using the original method of mean and standard deviation based CNR computation, the resulting ICC was negative, which is not theoretically possible. While negative ICC's or negative-ranging 95 % confidence intervals are observable based upon the data, it is recommended to simply interpret these values as representing poor reliability (Giraudeau, 1996). Use of the mode rather than the mean and standard deviation of the CC dramatically improved the reliability of the manual tracing method. The mode is more robust to extreme and infrequent values that may otherwise skew the data and contribute to large standard deviations. However, even when using the mode, the manually traced method still did not achieve the level of reliability seen in the template method. Based on this result, we attempted to unpack potential additional sources of variability. While prescribing manually traced masks on the data in native space has the advantage of reduced interpolation artifact, there is also the disadvantage that slight alterations in slice positioning can complicate the tracing procedure. For example, imperfect head positioning or rotation of the slice prescription, particularly a “roll” rotation along the y axis, can result in the SN appearing asymmetrical in the horizontal plane. This can make it more difficult to trace the SN, complicate the placement of reference circles in the CC, and could even contribute to signal intensity differences bilaterally. Similarly, the native space image retains relatively thick slices (2–2.5 mm), which requires the rater to make a difficult decision as to how far ventral the SN is clearly visible. In the template-defined pipeline, images are resliced to 1 mm isotropic and the pre-defined template minimizes this issue. Finally, after reviewing the processed manual tracing data, there were instances of significant eroding of the SN mask when using a threshold based on the CC standard deviation. The relatively few voxels remaining in the SN mask may then have contributed to increased variability over time and lower reliability. In contrast, the larger and more continuously defined CC region in the template-defined processing stream may have provided a less variable measurement, mitigating this issue.

As expected, relationships between NM signal and reality distortion symptoms were in the positive direction. When comparing manual tracing and template-defined variants that were most closely matched in terms of computational procedures (manual-tracing with mode and thresholded template-defined), relationships with reality distortion were quite similar (r = .47 for manual-mode and r = .45 for thresholded-template). Interestingly, both manual tracing and template-defined methods benefitted from adopting features from one another. Using the mode to compute CNR for the manual tracing methodology revealed a significant relationship to reality distortion symptoms that was previously obscured. Similarly, using thresholding procedures in the template-defined methodology strengthened the relationship to reality distortion symptoms from a modest correlation (r = .30, p = .24) to one that trended towards significance (r = .45, p = .06). Both methods did not show any relationship with poverty symptoms, which would be consistent with NM signal largely being linked to symptoms that are considered to be associated with the elevated dopamine, such as hallucinations and delusions. However, the mode-based manual tracing method also showed a significant relationship with disorganized symptoms, such as attention and positive formal thought disorder. While symptom scales are highly correlated, this pattern of results suggests that the thresholded template-defined method may offer greater specificity to reality distortion symptoms, although this finding should be considered preliminary. These correlations with reality distortion symptoms identified in the present study are generally in line with those observed in prior studies with larger samples (Cassidy et al. (2019) = .38, Wengler et al. (2024a) = .35). Importantly, other work has found substantial regional variability in the SN, as particular regions show stronger relationships with psychotic symptomatology than others (Cassidy et al., 2019). While not explored in the present manuscript, voxelwise analyses may offer additional clinical utility in identifying subregions that are more significantly related to clinical symptomatology. As a whole, in addition to our conclusions about the robustness of the template-defined versus manual tracing method for ROI definition, our results are also consistent with the dopaminergic hypothesis of schizophrenia and other psychotic disorders, in which increased subcortical dopamine contributes to the manifestation of positive symptoms (Howes and Kapur, 2009).

Our study had several limitations. The sample size was relatively small, which resulted in limited power particularly for the symptom correlations. The range of test-retest interval was also relatively wide, which could increase the variability of NM measurements. The present study also does not explore how treatment resistance or use of antipsychotic medications impact NM values or relationships with symptoms, which would be of value for future studies. One of the researchers who processed the data (JPR) was responsible for a larger percentage of subjects than the second rater (SP). While both were trained to the same level of reliability, SP's academic appointment ended at an earlier date and she necessarily analyzed fewer participants. This concern is mitigated by the fact that both timepoints and processing streams were always conducted by the same rater for any given subject. Ultimately, given that the focus of the study is in comparing two techniques of processing, the majority of these limitations would be expected to impact both strategies equally.

In conclusion, the results of our study suggest that a template-defined method for ROI-based NM analysis is preferred over manual tracing. We suggest that this method be incorporated as part of a standardized pipeline for NM analyses in future studies.

## CRediT authorship contribution statement

**Tyler A. Lesh:** Writing – original draft, Methodology, Formal analysis, Visualization, Funding acquisition, Conceptualization. **Jason Smucny:** Writing – original draft, Funding acquisition, Visualization, Formal analysis. **Joshua P. Rhilinger:** Formal analysis, Writing – review & editing, Data curation. **Sarvenaz Pakzad:** Data curation, Writing – review & editing. **Guillermo Horga:** Writing – review & editing, Methodology, Software. **Cameron S. Carter:** Writing – review & editing, Funding acquisition, Resources, Conceptualization.

## Funding

This work was supported by funding awarded to CSC (NIMH: R01MH122139) and TAL/CSC (State of California Department of Cannabis Control: 65323).

## Declaration of competing interest

The authors have no conflicts of interest to disclose.

## Data Availability

Data will be made available on request.
