# Supplementary material for: Test-retest reliability of template-defined vs. manual tracing processing streams in neuromelanin-sensitive magnetic resonance imaging analysis
Source: Neuroimage Rep. 2025 Sep 26;5(4):100290. doi: 10.1016/j.ynirp.2025.100290 (PMC12509897; doi:10.1016/j.ynirp.2025.100290)
Supplement: Multimedia component 1 [file mmc1.docx]

**Supplementary Material**

**Supplementary Figure 1. Upper Left:** Scatterplot showing the relationship between test and retest neuromelanin (NM) contrast to noise ratio (CNR) for the template-defined method. **Upper Right:** Scatterplot showing the relationship between test and retest NM CNR for the template-defined method using additional thresholding. **Lower Left:** Scatterplot showing the relationship between test and retest NM CNR for the manual tracing method using the mean. **Lower Right:** Scatterplot showing the relationship between test and retest NM CNR for the manual tracing method using the mode. Individuals scanned using the Tim Trio (*n* = 7) are shown in blue, individuals scanned using the Prisma (*n* = 12) are shown in red, and individuals scanned using the Skyra (*n* = 3) are shown in teal.


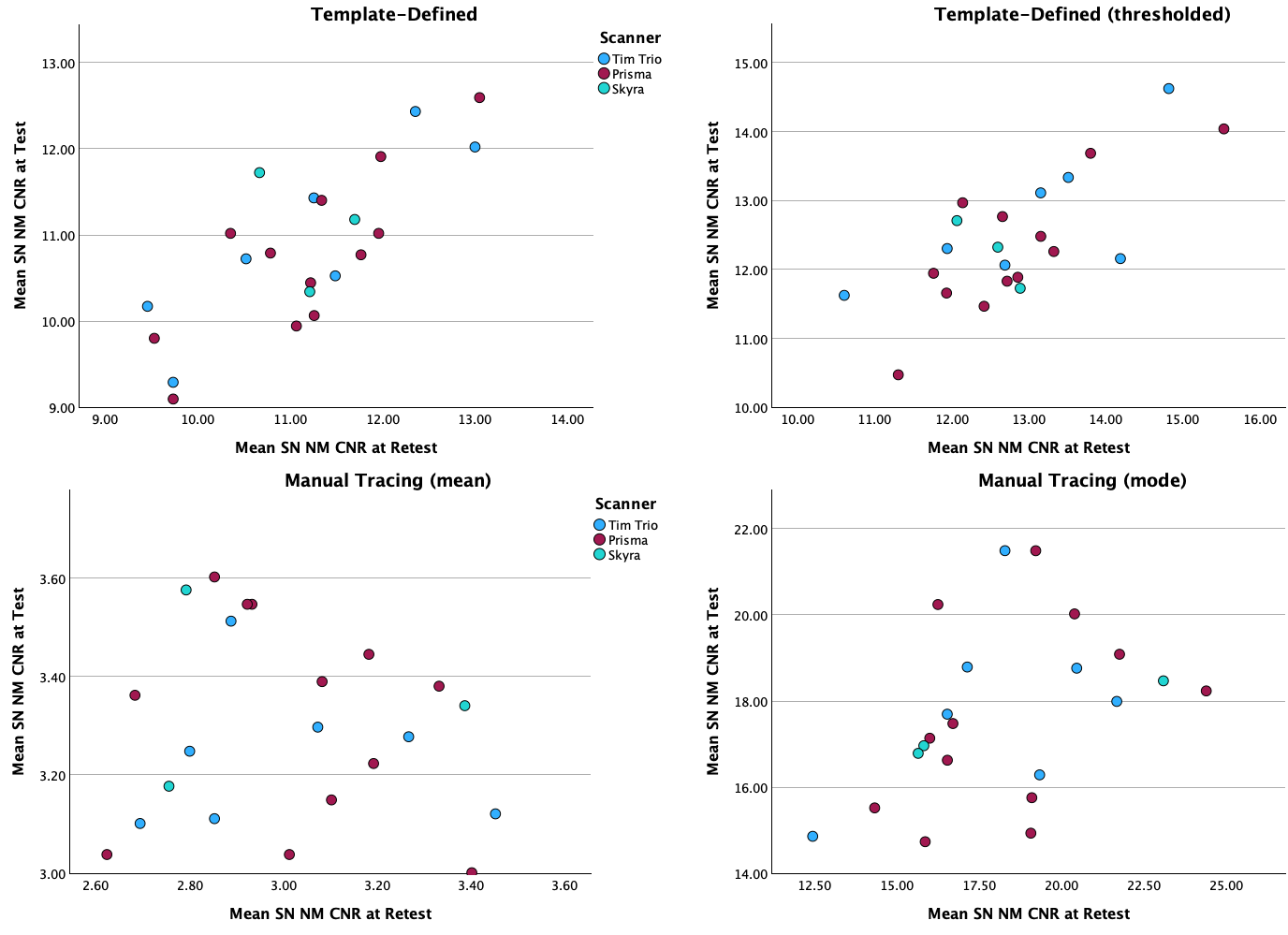


Supplementary Table 1:

|  | **Disorganization** | | **Reality Distortion** | | **Poverty** | |
| --- | --- | --- | --- | --- | --- | --- |
|  | r | p-value | r | p-value | r | p-value |
| **Method** |  |  |  |  |  |  |
| **Template-Defined** | .09 | .72 | .30 | .23 | -.09 | .73 |
| **Template-Defined (thresholded)** | .27 | .29 | .45 | .06 | .15 | .55 |
| **Manual Tracing** | -.19 | .45 | -.13 | .61 | .04 | .89 |
| **Manual Tracing (mode)** | .55 | .02 | .47 | .05 | .06 | .82 |

Correlations are Pearson’s r.
